# Synthesis of *Arapaima gigas* Growth Hormone (ag-GH) in HEK 293 Cells: Its Purification and Characterization via In Vivo Bioassay in Dwarf “Little” Mice

**DOI:** 10.3390/molecules31030572

**Published:** 2026-02-06

**Authors:** Eliana Rosa Lima, Jeniffer Cristina Ribeiro Melo, Filipe Menezes Bezerra, Miriam Fussae Suzuki, Amanda Palermo Nunes, Thais Cristina dos Anjos Sevilhano, João Ezequiel Oliveira, Riviane Garcez, Lucas Simon Torati, Geraldo Santana Magalhães, Cibele Nunes Peroni, Paolo Bartolini

**Affiliations:** 1 Institute of Energy and Nuclear Research (IPEN-CNEN), University City, São Paulo 05508-000, SP, Brazil; lima-eliana@hotmail.com (E.R.L.); jcrmelo00@gmail.com (J.C.R.M.); filipemenezes.sp@gmail.com (F.M.B.); mfsuzuki@alumni.usp.br (M.F.S.); mandis_1995_@hotmail.com (A.P.N.); thais_sevilhano@alumni.usp.br (T.C.d.A.S.); jeolivei@ipen.br (J.E.O.); cnperoni@ipen.br (C.N.P.); 2 Genetic Ichthyology Laboratory, Bioscience Institute, University of São Paulo, São Paulo 05508-090, SP, Brazil; riviane.silva@colband.com.br; 3 EMBRAPA Fisheries and Aquaculture, Água Fria Subdivision, Palmas 77008-900, TO, Brazil; lucas.torati@embrapa.br; 4 Immunopathology Laboratory, Butantan Institute, São Paulo 05503-900, SP, Brazil; geraldo.magalhaes@butantan.gov.br

**Keywords:** *Arapaima gigas*, growth hormone, pirarucu, HEK 293 cells, dwarf “little” mice, in vivo bioassay, phylogenetic analysis

## Abstract

*Arapaima gigas* growth hormone (ag-GH) cDNA was previously cloned from *A. gigas* pituitaries. In this work ag-GH has been synthesized using human embryonic kidney 293 cells (HEK293) transiently transfected with the 3.4-TOPO^®^ vector carrying ag-GH cDNA. The 4th day after transfection, the presence of putative ag-GH was detected via SDS-PAGE and Western blotting in comparison with human GH. Ion exchange purification exhibited a clearly symmetric peak, absent in the control medium. The purified fraction, submitted to high-performance size-exclusion chromatography (HPSEC), SDS-PAGE, and Western blotting, contained an immunoreactive molecule, slightly smaller than hGH as expected. MALDI-TOF-MS determined a high-resolution molecular mass of 21,220 Da versus a theoretical value of 21,150. A phylogenetic analysis positioned ag-GH within basal teleost lineages, consistent with earlier analyses of *A. gigas* gonadotrophic hormones, reinforcing the structural and functional conservation relevant for its biologic activity. An in vivo bioassay based on the body weight increase of dwarf “little” mice demonstrated a biological activity for ag-GH comparable to that of the international reference preparation of rec-hGH. For two species (*H. sapiens* and *A. gigas*) separated by an evolutionary period of >100 million years, such a positive biological correlation is remarkable.

## 1. Introduction

*Arapaima gigas*, a giant Amazonian fish of the order Osteoglossiformes, is very important for human nutrition but in danger of disappearing because of exploitation by predatory fishing [1,2,3]. Its commercial breeding, moreover, is quite limited, in large part because of its reduced reproduction in captivity [4,5,6,7,8,9]. This reduced reproductive output results from the biological and ecological characteristics of *A. gigas*. The species follows a K-selected life-history strategy, characterized by the production of a small number of large, yolk-rich eggs and intense parental care (mouth-brooding and nest guarding). Although this strategy enhances juvenile survival, it limits overall fecundity per spawning cycle. In addition, reproduction in *A. gigas* depends on specific environmental cues—such as seasonal flooding, temperature, and photoperiod—which restrict successful spawning both in the wild and under captive conditions.

Besides the pituitary gonadotrophic hormones that regulate the reproductive processes, follicle-stimulating hormone (FSH) and luteinizing hormone (LH), growth hormone (GH) has also been found to have immune and reproductive functions, stimulating spermatogonial proliferation and accelerating the spermatogenic processes in fish [10,11,12]. In addition, growth hormone exerts well-known effects on somatic growth and muscle mass increase. For these reasons, we are currently collaborating with established *A. gigas* reproduction centers in order to perform molecular cloning, characterization and practical application of ag-GH cDNA and ag-GH. Several types of GH have been cloned and synthesized in various fish species, but, as far as we know, none from the order Osteoglossiformes. Most of these recombinant GH, representing at least 10 different species, have been produced in *E. coli* (n = 17 references), two of them in yeasts [13,14], one in insect cells [15], one in HEK293 cells [16], and one in leaves of transgenic plants [17].

Our research group was responsible for setting up an in vivo bioassay in dwarf “little” mice, the results of which were perfectly comparable to the classic bioassay in hypofisectomized rats, thus avoiding a highly invasive, costly and time-consuming procedure [18]. This assay measures parameters of in vivo linear growth in an animal model closely related to isolated hGH deficiency type 1B. It has been widely applied to numerous bioassays for hGH and mGH potency determination. Interesting applications also include the exogenous stimulation of “little” mice with growth hormone releasing-peptide-2, as a reference assay for the standardization of a new assay for hGH determination in serum [19] and for the potency determination of a newly modified hGH polypeptide [20]. Finally, the dwarf “little” mice assay was also found to be useful for hGH determination in seeds of transgenic plants [21] and for PASylated hGH, with extended half-life in plasma, where PAS corresponds to the tripeptide Pro-Ala-Ser [22]. In a recent review, the lit/lit mice bioassay was cited as one of the best in vivo models for testing the biological activity of human GH preparations [23].

In addition to extensive physicochemical and immunological characterization, we conducted a phylogenetic analysis of GH sequences to define the evolutionary relationships of ag-GH within teleost lineages better. This analysis clearly positioned *A. gigas* among basal teleosts, consistent with previous findings on its gonadotropic hormones [24,25]. This evolutionary insight highlights conserved structural and functional elements essential for GH bioactivity and facilitates comparative endocrinological research, providing a scientific basis for further exploring its biological properties through our established lit/lit mouse bioassay. Therefore, the aim of this study was to obtain recombinant *A. gigas* growth hormone (ag-GH) using a mammalian expression system and to characterize its structural and biological properties. We hypothesized that, despite the large evolutionary distance between teleosts and mammals, conserved structural motifs in the GH molecule would allow cross-species receptor recognition and biological activity. This work provides the first demonstration of a recombinant GH from an Osteoglossiformes species expressed in mammalian cells and functionally active in a mammalian model, addressing a relevant gap in the understanding of GH evolution and expanding its potential biotechnological applications.

## 2. Results and Discussion

### 2.1. Synthesis of Arapaima gigas Growth Hormone via Transient Transfection of HEK293 Cells with the agGH-cDNA^TM^ 3.4-TOPO^®^ Vector

Employing the *A. gigas* GH cDNA sequence obtained by our research group in previous work [26], recombinant ag-GH was synthesized and fully characterized for the first time in HEK293 host cells, which are a quite unusual choice for a fish GH synthesis. This was primarily due to the fact that the synthesis in either the cytoplasm or periplasmic space of *E. coli* proved to be quite difficult, due primarily to the insufficient yield of a soluble protein. As far as we know, only *Anguilla japonica* GH, a phylogenetically related form of GH used for promoting somatic growth in eel larvae has been produced in HEK293 cells [16]. It is noteworthy that the ag-GH mature form, which consists of 185 amino acids, has a 44.7% amino acid identity with hGH and a calculated molecular mass of 21,150 Da versus a hGH molecular mass of 22,129 Da. A direct amino acid sequence alignment between the mature forms of ag-GH and hGH illustrates the distribution of conserved and divergent residues, including fully conserved cysteines and functionally relevant regions (Supplementary Figure S1), as previously characterized through sequence alignment and structural analyses [26].

To ensure that the observed protein expression and downstream analyses were specifically attributable to ag-GH production, a negative control transfection and cultivation were performed in parallel, in which the only difference was the absence of ag-GH cDNA in the expression pcDNA^TM^ 3.4-TOPO^®^ vector.

### 2.2. Qualitative Analysis of HEK293 Conditioned Media

**Figure 1:**
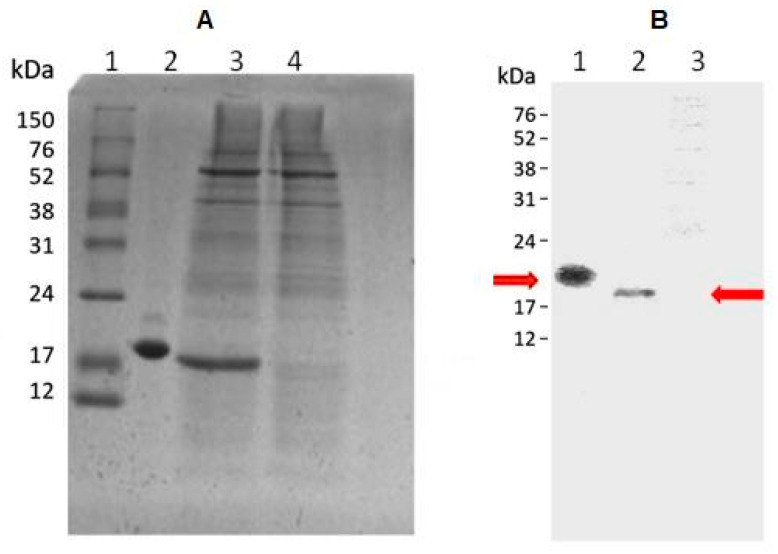
(**A**) SDS-PAGE 15% under non-reducing conditions. Lane 1: Molecular weight marker (bands at 22, 21 kDa, etc.); Lane 2: Positive control, recombinant hGH (1 µg, apparent Mw ≈ 22 kDa); Lane 3: Recombinant ag-GH from HEK293F transfection (4th day, apparent Mw ≈ 21 kDa); Lane 4: Negative control (TOPO^®^ vector without ag-GH cDNA). (**B**) Western blot probed with anti-rat-GH antiserum. Lane 1: Positive control hGH (1 µg, apparent Mw ≈ 22 kDa); Lane 2: Recombinant ag-GH (apparent Mw ≈ 21 kDa); Lane 3: Negative control. arrows indicate GH bands. Apparent molecular weights were estimated relative to the migration of the protein marker bands.

Conditioned medium from HEK293 cells transiently transfected with agGH-cDNA inserted into the pcDNA^TM^ 3.4-TOPO^®^ vector and the negative control medium, collected four days post-transfection, were analyzed via SDS-PAGE and Western blotting. In the SDS-PAGE analysis under non-reducing conditions (Figure 1A), an intense band corresponding to recombinant ag-GH was observed in lane 3, migrating slightly faster than human GH (hGH, lane 2), suggesting a lower molecular weight for ag-GH. No band was detected in the corresponding negative control lane (lane 4). Although the calculated molecular masses of hGH and ag-GH differ by approximately 1 kDa, their apparent electrophoretic mobilities differ more noticeably, which likely reflects sequence-dependent anomalous migration, a well-recognized limitation of SDS-PAGE for small and structurally compact proteins. Western blot analysis (Figure 1B), using anti-rat GH affinity-purified igG, confirmed the immunoreactivity of recombinant ag-GH, with bands clearly observed only for hGH and ag-GH. This cross-reactivity is consistent with the 57.3% amino acid identity previously reported between ag-GH and rat-GH [26]. As expected, the negative control showed no detectable immunoreactivity.

### 2.3. Ion Exchange Purification on QFF-Sepharose of HEK293F Conditioned Media

**Figure 2:**
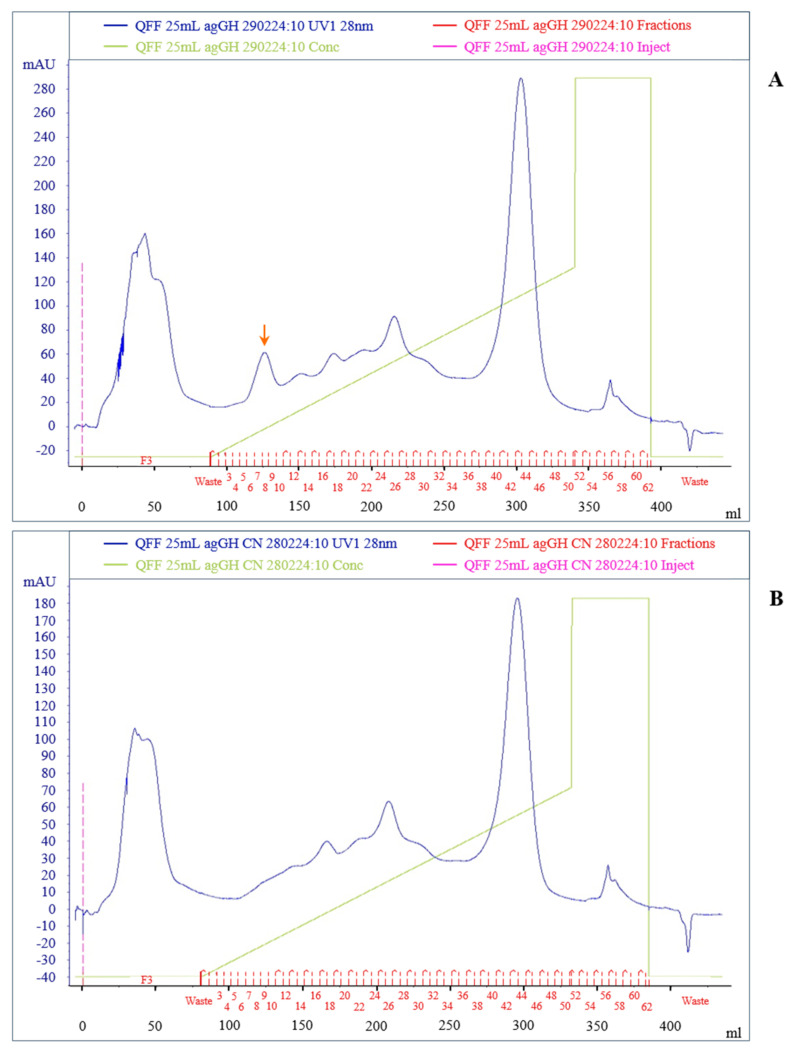
Ion exchange purification on QFF-Sepharose of HEK293F conditioned medium. (**A**) Fourth-day conditioned medium production from cells transfected with the 3.4-TOPO^®^ vector containing ag-GH cDNA. (**B**) Negative control: conditioned medium obtained using the same expression vector lacking the ag-GH cDNA insert. Chromatograms were generated using identical purification conditions. The arrow in panel A highlights a symmetrical peak detected exclusively in fractions 7–9 (pool A), which was absent in the negative control and subsequently identified as ag-GH.

The QFF-Sepharose purifications of the HEK293 conditioned medium, transfected with the agGH-cDNA-3.4-TOPO^®^ vector, and of the negative control medium are shown in Figure 2. The ion exchange separation was carried out according to a protocol used for the purification of the hGH receptor antagonist G120R-hGH [27]. Two almost identical chromatograms were obtained, with the exception of a symmetrical peak appearing only in fractions 7-8-9 of the positive medium, indicated by an arrow. This peak presumably corresponded to ag-GH, as demonstrated by a series of confirmatory analyses.

### 2.4. HPSEC Analysis of the Fractions Obtained After QFF-Sepharose Purification

**Figure 3:**
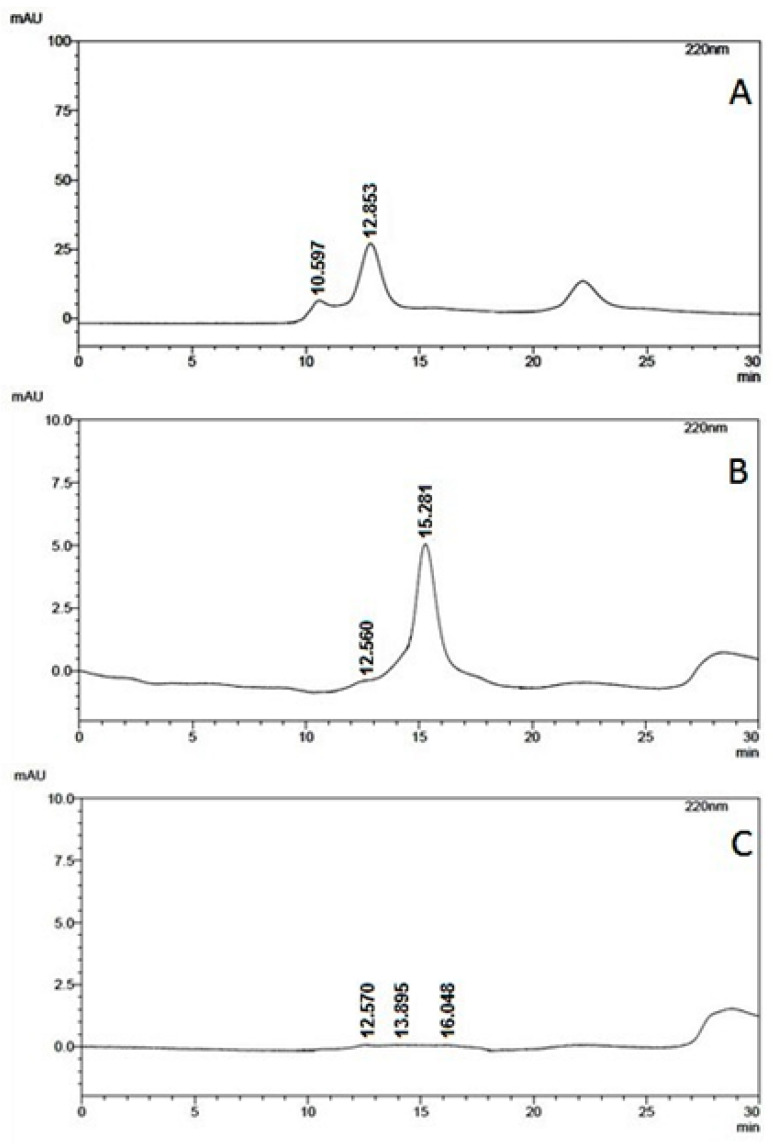
HPSEC analysis of: (**A**) rec-hGH, 10 μg/10 μL; (**B**) “pool a” from the QFF-Sepharose purification (100 µL); (**C**) fraction 8 from the QFF purification of the negative control (100 µL).

As shown in Figure 3, in comparison with hGH (retention time, t_R_ = 12.853 min), the fraction derived from the agGH-cDNA-3.4-TOPO^®^ vector presents a main peak (t_R_ = 15.281 min) with just a small amount of dimer (t_R_ = 12.560), indicating again that the molecular mass of the putative ag-GH is smaller than that of hGH. As expected, the negative control conditioned medium did not present any significant peaks (Figure 3C).

**Figure 4:**
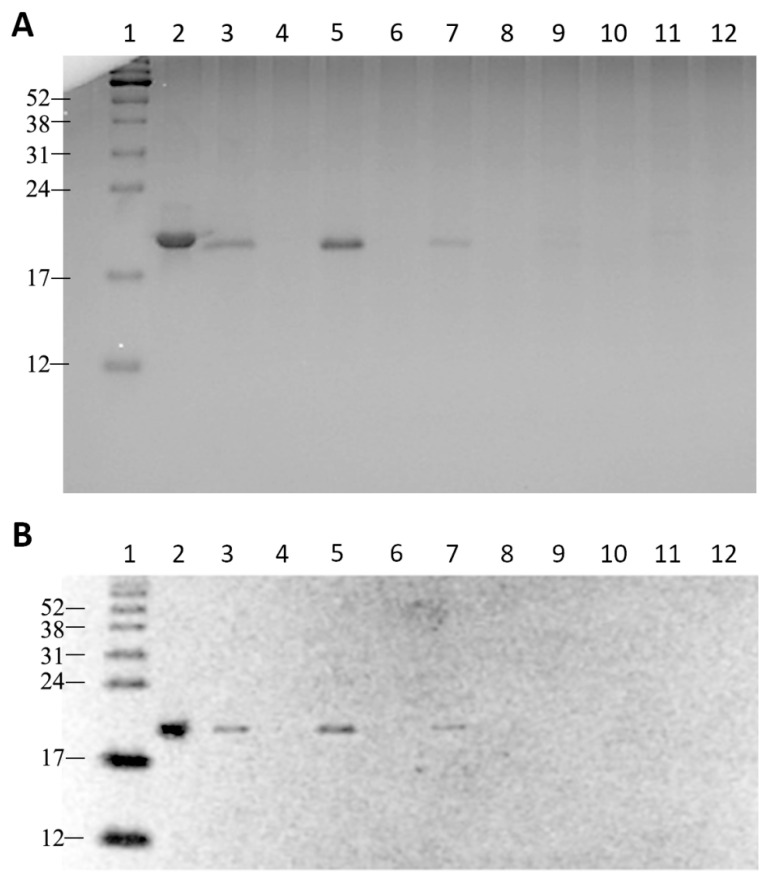
(**A**) SDS-PAGE run under non-reducing conditions: 1. Molecular weight ladder; 2. hGH, 1 µg/2 µL; lanes 3, 5, 7, 9, 11 correspond to fractions 6, 8, 10, 12, 14 from the ag-GH QFF purification (20 µL); lanes 4, 6, 8, 10, 12 correspond to fractions 6, 8, 10, 12, 14 from the negative control QFF purification (20 µL). (**B**) Western blot, using anti-rat-GH antiserum, of the same fractions analyzed in (**A**).

The confirmation of the presence of a GH isoform in the QFF-Sepharose eluted fractions comes from Figure 4, which presents a complete screening of the fractions obtained after QFF-Sepharose purification of the positive conditioned medium and the negative conditioned medium via SDS-PAGE or Western blotting.

### 2.5. Qualitative and Quantitative Analysis of Purified ag-GH

The HPSEC chromatogram of ag-GH from “pool a” of QFF-Sepharose after purification, shown in Figure 3, exhibits a symmetrical peak with a t_R_ = 15.281 min for ag-GH, as compared to t_R_ = 12.853 min for hGH (Figure 3A). From a quantitative point of view, based on the hGH area (10 µg) we calculated that the ag-GH peak in Figure 3B contained 25 µg, corresponding to a total yield of 18.7 mg/L for the whole HEK293 production. Although rather small, this production was about the same order as that obtained for ag-FSH synthesis (~28 mg/L) in the same HEK293 cells [25].

### 2.6. MALDI-TOF-MS Analysis of Purified ag-GH Versus the International Reference Preparation of rec-hGH NHPP

**Figure 5:**
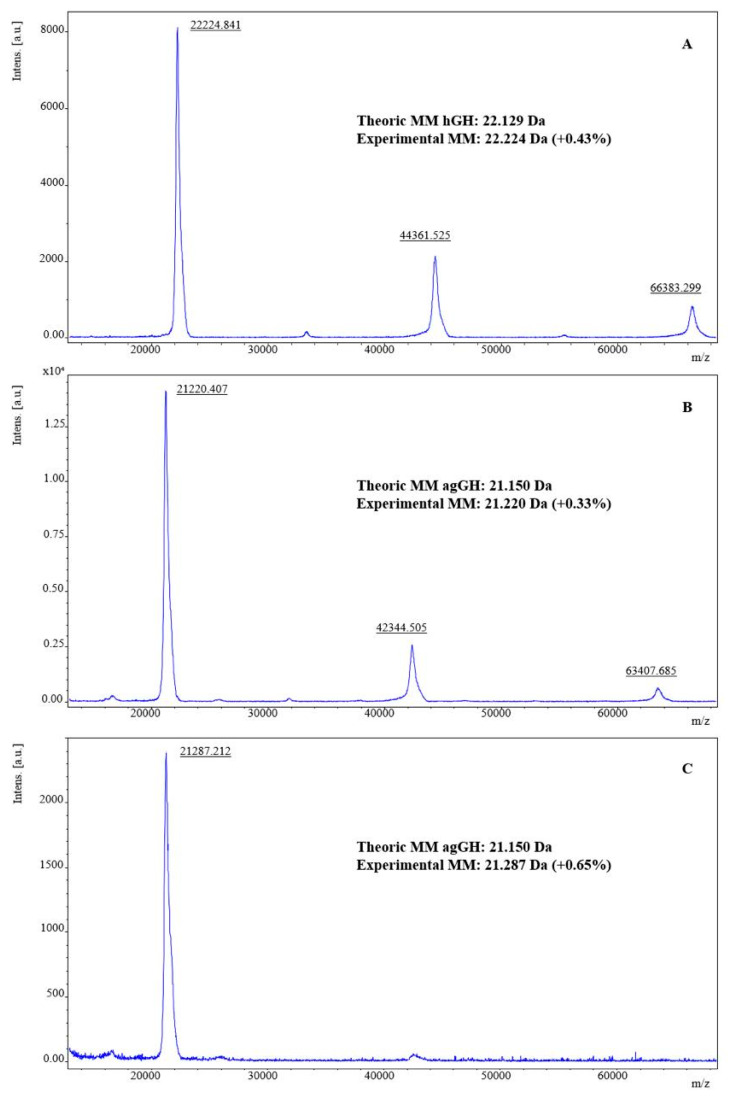
MALDI-TOF-MS analysis of: (**A**) hGH reference preparation from the National Hormone and Pituitary Program (NHPP); (**B**) purified ag-GH dissolved in 0.025 M (NH_4_)HCO_3_ and lyophilized; (**C**) purified ag-GH dissolved in 0.025 M (NH_4_)HCO_3_ and kept frozen at −20 °C. MM = molecular mass.

An accurate MALDI-TOF-MS analysis of purified ag-GH, compared with the international reference preparation of rec-hGH from the National Hormone and Pituitary Program, leaves little doubt as to the identity of this hormone (Figure 5). The differences between the experimental and the calculated theoretical values ranges from 0.33% to 0.65%. A comparison between a lyophilized (Figure 5B) and a merely frozen preparation (Figure 5C) also showed that, as frequently happens, lyophilization produced a significant amount of dimer and trimer, in which was also the case for the lyophilized international reference preparation.

When using the MM obtained from the dimer or trimer to calculate the experimental error in hGH or ag-GH determination, the error decreased from 0.43% to 0.23% to 0.0054%, and from 0.33% to 0.10% to 0.067%, respectively. Therefore, employing the MM of the trimer can significantly enhance the accuracy of our analysis. A single, well-defined peak of trimer, obtained via MALDI-TOF-MS and divided by 3, matched the theoretical MM of ag-GH (21,150 Da), providing an experimental value of 21,136 Da, i.e., with an experimental error of only 0.067%.

### 2.7. Phylogenetic Analysis of Arapaima gigas Growth Hormone (GH)

**Figure 6:**
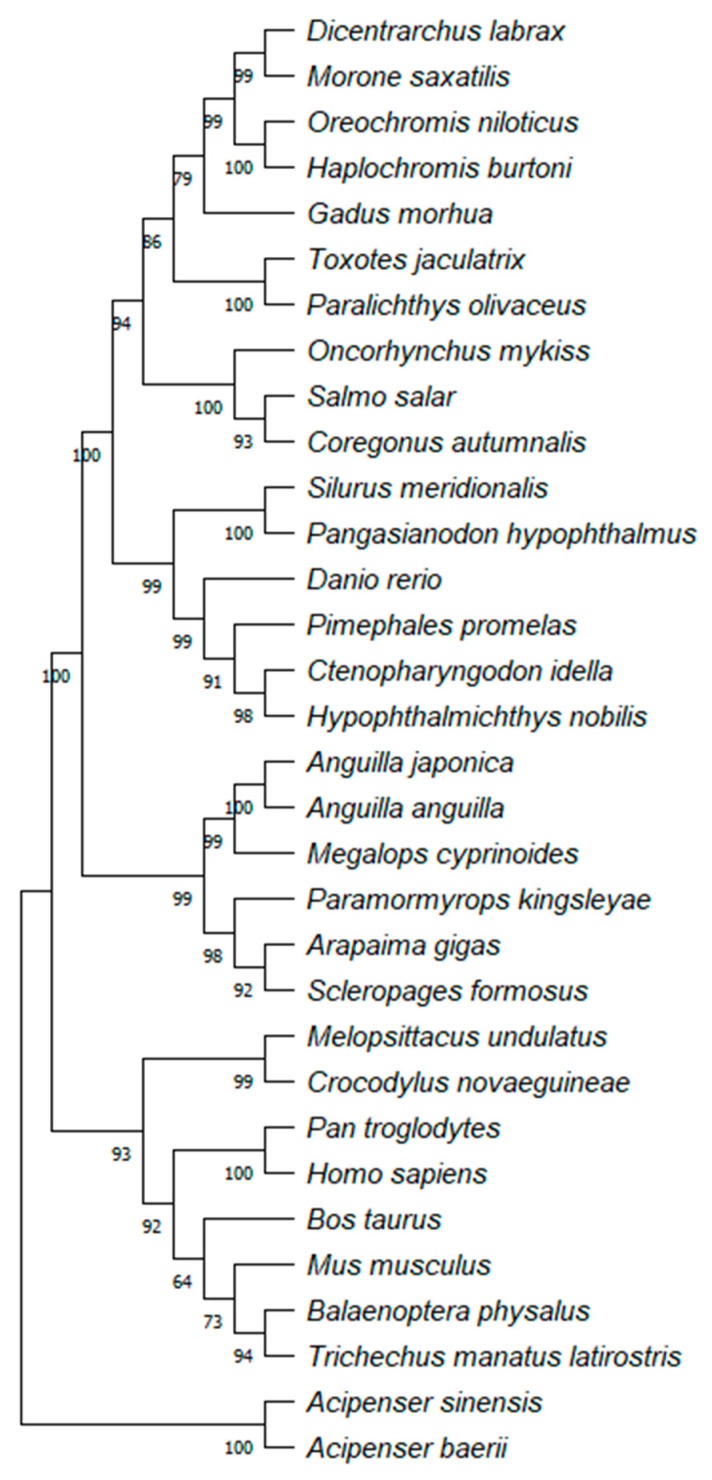
Phylogenetic tree of growth hormone (GH) sequences from *Arapaima gigas* and other vertebrate species. Neighbor-Joining distance tree based on Poisson-corrected amino-acid distances; 1000 bootstrap replicates (values shown at nodes). Alignment length: 222 positions after pairwise deletion; 32 taxa. Built in MEGA11.

The Neighbor-Joining (NJ) distance tree in Figure 6 summarizes sequence-similarity clustering among growth hormone (GH) amino-acid sequences from *Arapaima gigas* and representative vertebrates. In this NJ topology, *A. gigas* groups with basal teleost lineages (Osteoglossiformes), in line with prior hormone analyses from this species. The phylogenetic analysis of growth hormone (GH) sequences from *Arapaima gigas* aligns closely with the insights presented by Sevilhano et al. [25] and Faria et al. [24], who analyzed the β-subunits of follicle-stimulating hormone (FSHβ) and luteinizing hormone (LHβ) and the α-subunit of gonadotropic hormones (GTHα), respectively. Employing NJ methods with robust bootstrap support, the GH phylogeny situates *A. gigas* within basal teleost lineages, specifically the Osteoglossiformes order. This placement corroborates the findings of both Sevilhano et al. and Faria et al., which similarly identified a basal teleost position for *A. gigas*. In these earlier studies, significant amino acid sequence conservation was found between *A. gigas* and ancient groups such as Acipenseriformes, Anguilliformes, and Ostariophysi.

The Neighbor-Joining (NJ) distance tree, constructed using Poisson-corrected amino-acid distances and 1000 bootstrap replicates, is shown in Figure 6. Branch support values are indicated below nodes. The analysis included 32 sequences (222 aligned positions after pairwise deletion) and was carried out in MEGA 11. The node supporting the grouping of *A. gigas* with *Scleropages formosus* (both Osteoglossiformes) showed a bootstrap value of 92%, indicating strong support for this relationship. The adjacent node linking this Osteoglossiform clade to the basal teleost *Paramormyrops kingsleyae* had a bootstrap value of 98%, further confirming the robust placement of *A. gigas* among early-branching teleost lineages.

This consistent placement of *A. gigas* within basal teleost lineages suggests that the growth hormone (GH) gene has undergone a relatively slower rate of molecular evolution in early-branching actinopterygians. The high amino-acid sequence conservation observed between *A. gigas* and other ancient groups (e.g., Acipenseriformes and Anguilliformes) indicates that key structural and functional domains of GH have remained strongly conserved throughout vertebrate evolution. This pattern likely reflects the preservation of ancestral motifs essential for receptor binding and endocrine regulation, consistent with the conserved physiological roles of GH across vertebrate taxa.

### 2.8. In Vivo Bioassay in Dwarf “Little” Mice

**Figure 7:**
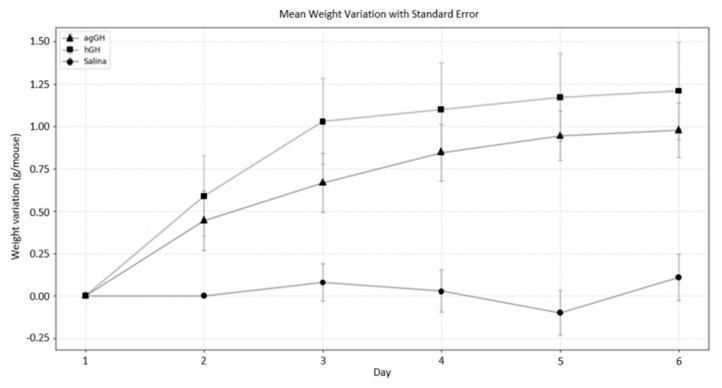
Six-day in vivo bioassay in dwarf “little” mice (n = 10 per group). The curves represent groups treated daily with human growth hormone (hGH), *Arapaima gigas* growth hormone (ag-GH), or saline (negative control). The equations for each group are as follows: negative control, Y = 0.0057X (r = 0.145; not significant); hGH, Y = 0.231X + 0.021 (r = 0.900; *p* < 0.02); agGH, Y = 0.185X + 0.012 (r = 0.932; *p* < 0.01). Data points indicate mean weight variation, and vertical error bars represent the standard error of the mean (SEM) for each group at each time point. Group comparisons were conducted using one-way ANOVA followed by pairwise unpaired Student’s *t* tests at each experimental day, with Bonferroni correction for multiple comparisons (α = 0.0167). Significant differences (*p* < 0.05) were observed between hGH and negative control, and between ag-GH and negative control from Day 2 onward. No significant differences were detected between hGH and ag-GH on any day (*p* > 0.05). ANOVA indicated overall group differences from Day 2 onward, consistent with results of pairwise comparisons between treated groups and the control. According to a slope-ratio analysis the bioactivity of ag-GH versus hGH was calculated as approximately 80%.

The final proof of ag-GH identity and bioactivity was provided by an in vivo bioassay in dwarf “little” mice [18] (Figure 7). The importance of this assay is due not only to the demonstration of the growth promoting activity of this hormone, but also in revealing its functional conservation across species separated by long evolutionary periods, as the assay involves *Arapaima gigas*, *Mus musculus*, and *Homo sapiens*. The corresponding amino acid identities are 57.8% between ag-GH and mouse-GH, 66.1% between mGH and hGH, and 44.7% between ag-GH and hGH.

In this evolutionary context, previously reported structural analyses provide a mechanistic background for the functional assessment performed here. In our earlier study [26], sequence alignment and three-dimensional modeling showed that ag-GH and hGH share the conserved four-helix bundle architecture, including full conservation of the cysteine residues forming the two disulfide bonds, with no major conformational differences despite their long evolutionary separation. Importantly, the most conserved regions overlapped with helices involved in growth hormone receptor binding, suggesting preservation of key structural determinants required for receptor interaction. The present in vivo bioassay therefore serves as a functional test of these prior structural predictions.

Although in vitro assays can provide higher-resolution and pathway-specific measurements of growth hormone receptor signaling, the in vivo dwarf (“little”) mouse bioassay remains a well-established and biologically integrative approach for assessing growth-promoting activity, as it reflects receptor engagement and physiological outcomes at the organismal level. The approximate value of “~80%” biological activity refers to a descriptive comparison of the slopes of the regression lines obtained in this assay and should not be interpreted as a formal relative potency estimate, as no parallel-line or slope-ratio analysis was performed.

The single-dose design employed in this study was intended to establish biological functionality rather than to perform a formal potency determination. While multi-dose studies and downstream biochemical markers such as serum IGF-1 levels can provide additional quantitative and mechanistic insights, the growth response observed in dwarf (“little”) mice is a well-established GH receptor-dependent endpoint that integrates ligand–receptor interaction and systemic endocrine signaling. Such analyses will be the subject of future dedicated studies.

The biological significance of these results lies in the noteworthy conservation of the growth hormone (GH)–receptor interaction across vertebrates. The ability of a teleost GH to promote somatic growth in a mammalian model implies that key structural elements responsible for receptor binding and activation are evolutionarily conserved. Although sequence identity between ag-GH and mouse- or human-GH is moderate, the preservation of critical contact residues within helices I and IV and the connecting loops of the four-helix bundle structure likely enables efficient recognition by the murine GH receptor. This structural compatibility explains the observed cross-species bioactivity and supports the concept of a highly constrained GH–GHR signaling system that has remained functional for over 100 million years of vertebrate evolution.

The observed somatic growth in dwarf “little” mice following ag-GH administration provides functional evidence that the recombinant teleost hormone interacts with the murine GH receptor (GHR). The GH–GHR mechanism—ligand binding, receptor dimerization, and downstream JAK2–STAT5 signaling—is highly conserved among vertebrates [28]. Teleost fishes possess two GHR types (GHR1 and GHR2), as shown by Jiao et al. [29], which differ in signaling dynamics; experiments in trout demonstrate that both receptor types can activate STAT5, ERK, and Akt pathways [30]. Although mammals have a single GHR ortholog, its ligand-binding domain retains structural homology to teleost GHRs, providing a plausible basis for cross-species recognition. The lit/lit mouse bioassay is well established as GHR-dependent, so the growth stimulation by ag-GH supports receptor-mediated activity in the mammalian system.

## 3. Materials and Methods

### 3.1. Synthesis of ag-GH in Human Embryonic Kidney 293 (HEK293) Cells

**Figure 8:**
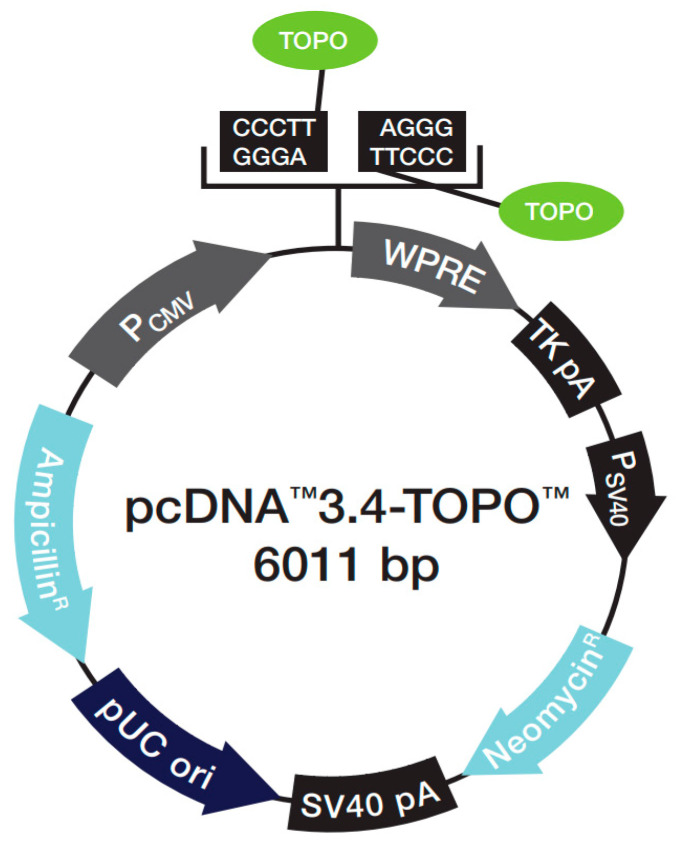
pcDNA^TM^ 3.4-TOPO^®^ vector to which the sequence of ag-GH cDNA was inserted.

The transient transfection and synthesis of ag-GH was performed via the Expi 293^TM^ Expression System Kit (Life Technologies, Carlsbad, CA, USA). The vector appropriate for HEK293 transient transfection was obtained from the commercial pcDNA^TM^ 3.4 TOPO^®^ vector (Thermo Fisher Invitrogen, Carlbad, CA, USA), into which the ag-GH cDNA was inserted via PCR reaction using primers forward 5′ GAG GCT TCT TAC TGG GCT CC 3′ and reverse 5′ AAG TGC AGT TGC TCT CCA CAA 3′, including the Kozak consensus sequence according to the kit instructions (pcDNA^TM^ 3.4 TOPO^®^ TA cloning kit) (Figure 8).

Ag-GH cDNA was inserted into the pcDNA^TM^ 3.4 TOPO^®^ vector and 30 µg of the obtained plasmid DNA were added to ExpiFectamine^TM^ 293 Reagent (Life Technologies, Carlsbad, CA, USA), in Opti-MEM^®^ medium. This solution was incubated for 20–30 min at room temperature, thus obtaining the DNA–ExpiFectamine^TM^ 293 Reagent complex (Life Technologies, Carlsbad, CA, USA). This mixture (3 mL) was then added to a 125 mL Erlenmeyer shaker flask containing 7.5 × 10^7^ Expi293^TM^ cells in Expi293^TM^ Expression medium, reaching a final volume of 28.5 mL. A negative control was also prepared using the same reagents and procedure, but without ag-GH cDNA insertion into the TOPO^®^ vector. Transfection was then carried out in a 37 °C incubator with a humidified atmosphere of 8% CO_2_ in air, using an orbital shaker rotating at 125 rpm. Eighteen hours after the addition of the DNA–ExpiFectamine™ complex to the HEK293 cell suspension, Transfection Enhancer 1 and Transfection Enhancer 2 were added to the 125 mL flask, bringing the total culture volume to 30 mL. Following enhancer addition, cells were maintained in culture under the same shaking and incubation conditions for five consecutive days. Small aliquots of conditioned medium were collected daily, from 48 h post-transfection onward, for expression analysis by SDS-PAGE and Western blotting, while the remaining culture was kept intact until the final harvest on day five.

### 3.2. SDS-PAGE and Western Blotting

Samples of the HEK293 conditioned media and their purified fractions were analyzed by 15% polyacrylamide gel electrophoresis (SDS-PAGE) under non-reducing conditions, as previously described, employing Coomassie Brilliant Blue G-250 for the staining [31]. Analyses were performed under non-reducing conditions to preserve native disulfide bonds and to assess the possible presence of disulfide-linked dimers or higher-order oligomers of ag-GH [18,32]. For Western blotting, the semi-dry transfer technique on nitrocellulose membrane was used, with anti-rat-GH affinity purified rabbit IgG (AFP5672099, NHPP, Torrance, CA, USA), 1:1000, and goat anti-rabbit IgG (Pierce Biotechnology, Rockford, IL, USA) conjugated to horseradish peroxidase (1:10,000). This antibody has been extensively validated for cross-species GH detection and was employed here solely for qualitative identification of recombinant ag-GH. Proteins were visualized with Luminata Forte (Merck, Burlington, MA, USA) and captured by the photodocumentation system (Uvitec Alliance 4.7, Cambridge, UK).

### 3.3. High-Performance Size-Exclusion Chromatography (HPSEC)

HPSEC was used for analytical purposes employing a G-2000 SW column (600 mm × 7.5 mm I.D.) particle size of 10 µm and pore size of 125 Å from Tosoh Bioscience (Montgomeryville, PA, USA), in a Shimadzu model SCL-10 A apparatus (Shimadzu Corporation, Kyoto, Japan). UV absorbance detection was at 220 nm, with a flow rate of 1.0 mL/min, using 0.15 M NaCl in 0.02 M sodium phosphate buffer, pH 7.0, as the mobile phase for isocratic elution.

### 3.4. Anion-Exchange Chromatography on Q-Sepharose Fast Flow (QFF)

HEK293 conditioned medium, from either positive or negative control, was applied to a QFF-Sepharose column (125 × 16 mm I.D.) equilibrated in 0.01 M ammonium acetate buffer, pH 7.5, under a linear gradient of 0–1 M NaCl in 0.01 M ammonium acetate buffer, pH 7.5, at a flow rate of 2 mL/min, using 10 column volumes (10 × 25 mL) [27]. The peak of putative ag-GH, absent in the negative control medium, was analyzed via HPSEC, SDS-PAGE and Western blotting.

### 3.5. MALDI-TOF-MS

The molecular mass of hGH (National Hormone and Pituitary Program-NHPP, USA) and of ag-GH, lyophilized or kept frozen in 0.025 M (NH_4_)HCO_3_, was determined using approximately 25 µg of each protein by MALDI-TOF-MS on an AutoFlex equipment (Bruker Daltonics, Bremen, Germany), operated in a linear positive ion mode. The analysis was carried out at the Biomass Laboratory-CEFAP of the University of São Paulo (USP), Brazil. A diluted protein sample (1:5; 1:10 and 1:20 from a 1 mg/mL solution) was mixed 1:1 with MALDI matrix solution (sinapinic acid, 7mg/mL in 0.1% trifluoracetic acid and 50% acetonitrile) and spotted directly onto the MALDI plate (1 µL). The analysis was performed in linear positive mode in the range from 10,000–70,000 Da.

### 3.6. Phylogenetic Analysis

The phylogenetic analysis was performed using MUSCLE for multiple sequence alignment of growth hormone (GH) amino acid sequences from *Arapaima gigas* and 31 other vertebrate species, including teleosts, reptiles, birds, and mammals. Sequences were retrieved from GenBank and ambiguous positions were removed using the pairwise deletion option. A total of 222 amino acid positions were retained for phylogenetic inference. The evolutionary relationships were inferred using the Neighbor-Joining (NJ) method [33] with bootstrap resampling (1000 replicates) [34] to assess branch support. Evolutionary distances were estimated using the Poisson correction model [35]. Acipenseriformes (*Acipenser baerii* and *Acipenser sinensis*) were set as the outgroup due to their basal position within Actinopterygii. The tree was constructed and visualized in MEGA11 [36], and its topology was compared with previous studies on GH evolution in teleosts. This Neighbor-Joining (NJ) analysis was used to depict overall sequence-similarity patterns (i.e., a distance-based clustering) and was not intended as a model-based phylogenetic reconstruction.

### 3.7. In Vivo Bioassay in Dwarf “Little Mice”

Little mice (C57BL/6J lit/lit) were obtained in our animal house from matings of C57BL/6J lit/lit males to C57BL/6J lit/+ females. Dwarf little mice were used when their average body weight was approximately 9.28 ± 0.78 g. The daily weight variation in the last 10 days (pre-assay) was within the limits of 0.0025 ± 0.0045 g/day [32]. This determined a rejection of about 10% of the population of homozygotic dwarf mice. The GH preparations were administered intraperitoneally daily in a volume of 100 µL containing 10 µg of each hormone. The experimental groups included recombinant *A. gigas* GH (ag-GH), the international reference preparation of recombinant human GH (NHPP, USA) as a positive control, and saline as a negative control.

The six-day assay duration is consistent with the validated lit/lit mouse bioassay used for GH potency determination, which reliably detects growth-promoting effects within this timeframe. Body weight was recorded daily, and individual weight variation was expressed as grams gained per mouse (g/mouse). Food and water were provided ad libitum and were identical across groups. In the lit/lit model, spontaneous growth in untreated controls is negligible; thus, increases in body weight after GH treatment represent true somatic growth rather than changes in feeding behavior or hydration [18]. This procedure was approved by the local animal ethics committee (CEUA/IPEN 61/24, 25 April 2024).

### 3.8. Statistical Analyses

Quantitative variables, reported as mean ± SD, were analyzed using unpaired Student’s *t* tests for pairwise comparisons between groups (agGH vs. hGH, agGH vs. Saline, and hGH vs. Saline) on each experimental day. One-way ANOVA was applied to evaluate overall differences among the three groups simultaneously. Growth equations were generated by fitting the data to a linear relationship. The linear and independent coefficients, calculated for each experimental group, were obtained using standard Python libraries. Prior to applying parametric tests, data were checked for normality (Shapiro–Wilk test) and for homogeneity of variance (Levene’s test). These tests confirmed that assumptions of normal distribution and equal variances were met. Statistical significance was defined as *p* < 0.05. Statistical and graphical analyses were performed using Python (version 3.10) with the libraries NumPy, Pandas, SciPy, Statsmodels, and Matplotlib.

## 4. Conclusions

*A. gigas* GH was synthesized for the first time, yielding 187 mg/L in HEK293 cells. As far as we know, among fish, only *Anguilla japonica* GH has also been produced in HEK293 cells. The identity, quality, and quantity of ag-GH were confirmed via SDS-PAGE analysis, Western blotting, HPSEC, and MALDI-TOF-MS. Remarkably, the in vivo bioassay, based on the body weight increase in dwarf “little” mice, demonstrated that ag-GH showed a growth-promoting response comparable (~80%) to that elicited by the NHPP-AFP8990 (a reference preparation of recombinant hGH) despite the evolutionary divergence of more than 100 million years between *A. gigas* and *H. sapiens*. Additionally, the phylogenetic analysis confirmed the position of ag-GH among basal teleost lineages, underscoring the structural and functional conservation responsible for this significant biological activity.

While GH sequences typically display great variability due to their diverse biological roles, the notable conservation among *A. gigas* and closely related basal teleosts underscores the strong evolutionary constraints associated with essential physiological functions, including growth regulation and metabolic control. These phylogenetic insights emphasize the biological and biotechnological importance of GH from *A. gigas*, particularly its potential in aquaculture for endocrinological and aquaculture applications such as selective breeding, growth optimization, and stress resistance studies. The consistent evolutionary findings from GH and gonadotropic hormone analyses reinforce the importance of the role that *A. gigas* hormones can play in the conservation and sustainable management of this ecologically and commercially important Amazonian fish species.

## Data Availability

The original contributions presented in this study are included in the article/Supplementary Material. Further inquiries can be directed to the corresponding author.

## Supplementary Materials

The following supporting information can be downloaded at: https://www.mdpi.com/article/10.3390/molecules31030572/s1, Figure S1: Amino acid sequence alignment between the mature forms of *Arapaima gigas* growth hormone (ag-GH) and human growth hormone (hGH).

## Abbreviations

The following abbreviations are used in this manuscript:

ag-GH	*Arapaima gigas* Growth Hormone
HEK293	Human Embryonic Kidney 293 cells
SDS-PAGE	Sodium Dodecyl Sulfate Polyacrylamide Gel Electrophoresis
HPSEC	High-Performance Size-Exclusion Chromatography
MALDI-TOF-MS	Matrix-Assisted Laser Desorption/Ionization Time-of-Flight Mass Spectrometry
NHPP	National Hormone and Pituitary Program
QFF	Q-Sepharose Fast Flow
PCR	Polymerase Chain Reaction
FSHβ	Follicle-Stimulating Hormone β Subunit
LHβ	Luteinizing Hormone β Subunit
GTHα	Gonadotropic Hormone α Subunit
NJ	Neighbor-Joining (Phylogenetic Method)
CO_2_	Carbon Dioxide
