# Supplementary material for: Synthesis of Arapaima gigas Growth Hormone (ag-GH) in HEK 293 Cells: Its Purification and Characterization via In Vivo Bioassay in Dwarf “Little” Mice
Source: Molecules. 2026 Feb 6;31(3):572. doi: 10.3390/molecules31030572 (PMC12899748; doi:10.3390/molecules31030572)
Supplement: Supplementary file 1 [file molecules-31-00572-s001.zip › molecules-4080880-supplementary.pdf]

Supplementary material

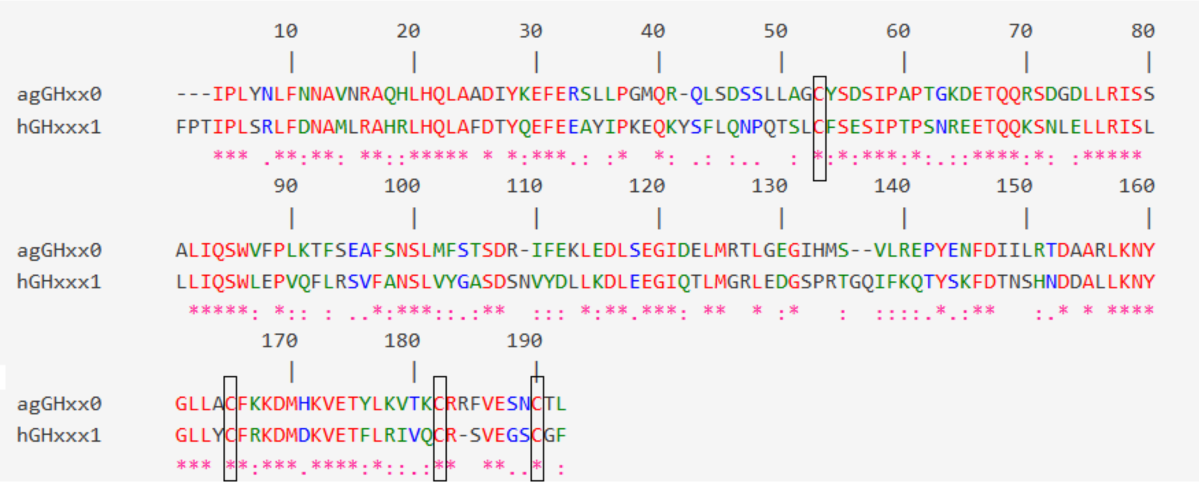

Figure S1: Amino acid sequence alignment between the mature forms of Arapaima gigas growth hormone (ag-GH) and human growth hormone (hGH).

Signal peptides were excluded prior to alignment. The ag-GH and hGH sequences correspond to GenBank accessions OP575308.1 and MT321110.1, respectively. The alignment was generated using Clustal Omega, and identical amino acids represent 44.79% of the aligned positions. Conserved cysteine residues are indicated by rectangles. Putative growth hormone receptor–binding residues, previously identified by structural modeling and molecular dynamics analyses, include Lys41, Gln46, Ser51, Glu56, Ser62, Lys64, Arg167, and Lys168 in hGH, and the structurally corresponding residues Arg38, Asp42, Ala47, Asp52, Thr58, Arg60, Lys160, and Lys161 in ag-GH; these residues can be readily located based on the residue numbering shown (Lima et al., 2023).
